# Assessment of Aerobic Exercise Adverse Effects during COPD Exacerbation Hospitalization

**DOI:** 10.1155/2017/5937908

**Published:** 2017-02-06

**Authors:** Caroline Knaut, Carolina Bonfanti Mesquita, Laura M. O. Caram, Renata Ferrari, Victor Zuniga Dourado, Irma de Godoy, Suzana Erico Tanni

**Affiliations:** Botucatu Medical School, Universidade Estadual Paulista (UNESP), Department of Internal Medicine, Pneumology Area, Botucatu Campus, Botucatu, SP, Brazil

## Abstract

*Introduction.* Aerobic exercise performed after hospital discharge for exacerbated COPD patients is already recommended to improve respiratory and skeletal muscle strength, increase tolerance to activity, and reduce the sensation of dyspnea. Previous studies have shown that anaerobic activity can clinically benefit patients hospitalized with exacerbated COPD. However, there is little information on the feasibility and safety of aerobic physical activity performed by patients with exacerbated COPD during hospitalization.* Objective.* To evaluate the effects of aerobic exercise on vital signs in hospitalized patients with exacerbated COPD.* Patients and Methods.* Eleven COPD patients (63% female, FEV1: 34.2 ± 13.9% and age: 65 ± 11 years) agreed to participate. Aerobic exercise was initiated 72 hours after admission on a treadmill; speed was obtained from the distance covered in a 6-minute walk test (6MWT). Vital signs were assessed before and after exercise.* Results.* During the activity systolic blood pressure increased from 125.2 ± 13.6 to 135.8 ± 15.0 mmHg (*p* = 0.004) and respiratory rate from 20.9 ± 4.4 to 24.2 ± 4.5 rpm (*p* = 0.008) and pulse oximetry (SpO_2_) decreased from 93.8 ± 2.3 to 88.5 ± 5.7% (*p* < 0.001). Aerobic activity was considered intense, heart rate ranged from 99.2 ± 11.5 to 119.1 ± 11.1 bpm at the end of exercise (*p* = 0.092), and patients reached on average 76% of maximum heart rate.* Conclusion.* Aerobic exercise conducted after 72 hours of hospitalization in patients with exacerbated COPD appears to be safe.

## 1. Introduction

Chronic obstructive pulmonary disease (COPD) is characterized by progressive airflow obstruction that is not fully reversible. It is associated with inhaling particles or toxic gases which causes an abnormal inflammatory response in the lungs; it is primarily induced by smoking [1, 2].

Exacerbation events are relatively common in disease progression. About 70% of exacerbations are caused by respiratory infection from bacteria or virus. Exacerbation is associated with increased disease symptoms and worse lung function, quality of life, and physical capacity. Literature states that mean period to recover previous functional characteristics is three months [3–6].

Hospitalization may be necessary in patients with rapidly deteriorating respiratory symptoms and treatment includes mechanical ventilation which is associated with a higher mortality risk. To reduce functional exercise capacity loss during a severe exacerbation, studies have been performed to evaluate the effects of lower limb muscle training [3, 7, 8]. Most have assessed the influence of anaerobic training. Troosters et al. (2010) showed improved lower limb muscle strength without increased systemic inflammation after muscle training in hospitalized COPD patients with exacerbation; the authors did not identify adverse clinical events during muscle training. Similarly, in a randomized study of 46 hospitalized COPD patients, Borges and Carvalho (2014) found improved leg strength and quality of life 30 days after discharge in the group who received upper and lower limb anaerobic training during hospitalization and did not observe adverse clinical effects during the training program. However, studies evaluating the adverse effects of aerobic activity in hospitalized patients with exacerbated COPD are scarce in literature. Tang et al. (2012) showed that combined aerobic anaerobic training in exacerbated COPD patients presented 13 adverse events, one with a serious arrhythmia event. However, we found no other studies that had investigated adverse effects related to aerobic training during hospitalization in exacerbated COPD patients.

Therefore, the aim of this study was to evaluate the effects of aerobic exercise on vital signs and symptoms in hospitalized exacerbated COPD patients.

## 2. Subjects

This clinical study is part of a randomized controlled trial including 11 hospitalized patients with exacerbation COPD from March 2012 to October 2014. Exacerbation was considered according to the GOLD (Global Initiative for Chronic Obstructive Lung Disease) [2] criteria. COPD was confirmed prior to hospitalization by spirometry using a bronchodilator where forced expiratory volume in one second/forced vital capacity was less than 0.70 (FEV1/FVC < 0.70) and severe exacerbation was considered when hospitalization was needed and changes in maintenance medication or the introduction of corticosteroids and/or antibiotics was needed. The exclusion criteria were a Glasgow score < 15, Borg dyspnea score > 7 [9], instable heart disease, limited mobility, hemodynamic instability, and mechanical ventilation.

**Figure 1:**
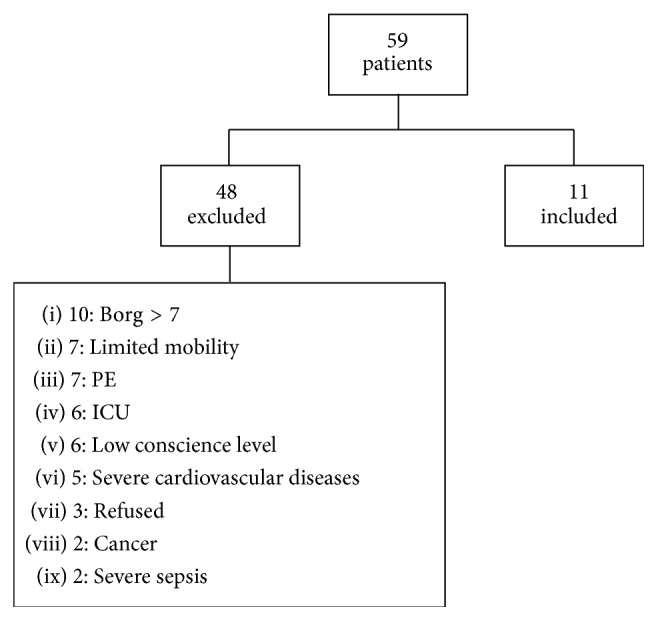
Patients evaluated for the study and exclusion criteria. PE: pulmonary embolism and ICU: intensive care unit.

This study was conducted at Botucatu Medical School Clinical Hospital, UNESP. All patients signed a free informed consent form and the study was approved by the institution ethics committee (Protocol 4027–2011) (Figure 1).

### 2.1. Study Design

All patients were evaluated 24 hours after hospitalization for demographic characteristics (age, gender, occupation, education, and monthly income), smoking history (pack year calculation, smoking status), Charlson index [10], and body composition. After 48 hours of hospitalization, all patients performed a 6-minute walk test (6MWT) and a new spirometry test, and BODE index was calculated [11]. Finally, after 72 hours of hospitalization patients underwent aerobic exercise on a treadmill for 15 minutes.

## 3. Methods

### 3.1. Spirometry

Spirometry was performed using a portable computerized pulmonary function system (FERRARIS KOKO, Louisville, CO, USA), according to the American Thoracic Society [12]. FVC and FEV1 were measured in litres (L), and the FEV1/FVC calculated. Measurements were obtained before and 20 minutes after being given a metered 400 *μ*g dose of fenoterol as a bronchodilator. The FVC and FEV1 values were also expressed as a percentage of reference values [13].

### 3.2. Body Composition

Body composition was assessed by anthropometry and bioelectrical impedance. Anthropometry: height and weight were determined using a Filizola® balance with the patient barefoot and wearing light clothes, and body mass index (BMI) was calculated [BMI = weight (kg)/height  (m)^2^]. Bioelectrical impedance: patients were instructed to empty their bladder, rest for thirty minutes, and remove all metal objects before measurements. Resistance was measured in the right side of the body according to Kyle et al., (2004) (BIA 101, RJL Systems, Detroit, MI, USA). Fat Free Mass (FFM) was estimated from an equation developed for patients with respiratory failure [14] and FFM index T [FFMI = FFM (kg)/height (m)^2^] was calculated. Nutritional depletion was defined when FFMI < 15 kg/m^2^ for women and <16 kg/m^2^ for men [15].

### 3.3. Six-Minute Walk Test

The six-minute walk test was performed according to the American Thoracic Society [16]. Patients with hypoxemia or who presented with pulse oximetry < 85% during the test were supplemented with oxygen according to medical prescription [17]. In this case, the physical therapist walked beside the patient pulling the portable cylinder trolley.

### 3.4. Intensity of Dyspnea and Leg Fatigue

We used the Modified Medical Research Council (MMRC) scale which graduates dyspnea from 0 to 4 in relation to daily effort [1], the higher the score the more severe the dyspnea.

The Borg scale is to gauge the patient's feeling of dyspnea from 0 to 10, the higher the score the greater the dyspnea intensity [18].

### 3.5. Aerobic Exercise

Aerobic exercise was performed using a treadmill (Inbramed Master) with the patient walking for 15 minutes. Speed was obtained from the 6MWD with distance divided by 360 s (6 minutes × 60 seconds) and the value obtained in m/s was transformed into km/h by multiplying by 3.6. The slope was increased by one point according to the Borg dyspnea scale ≤ 3 (moderate dyspnea) which was evaluated every five minutes during the exercise. Pulse oximeter (SpO_2_) and heart rate (HR) were monitored throughout the exercise. ECG was used to evaluate arrhythmia. The Borg scale for dyspnea and the legs and respiratory rate and blood pressure were monitored at the beginning and end of the exercise. There were two-minute warm-up and recovery using a lower speed and no inclination. Patients with hypoxemia SpO_2_ < 85% during training were supplemented with O_2_ according to medical prescription. Aerobic exercise will be considered safe if patients did not present with any clinical adverse event during or after exercise.

### 3.6. Statistical Analysis

Descriptive analysis of data was performed using Sigmaplot 12.0 (Systat Software, Inc., San Jose, CA, USA). The data are presented in tables, and continuous variables with normal distribution are expressed as mean values with standard deviations and continuous variables with nonnormal distribution as medians and quartiles.

The Paired* t*-test was used to compare continuous variables with normal distribution in the same group of patients at two different times (variables assessed before and after aerobic exercise).

## 4. Results

### 4.1. Patient Characteristics

For the composition of this study, we identified 59 hospitalized patients with a diagnosis of exacerbated COPD. Of these, 48 (69%) were excluded for reasons already detailed above (Figure 1). Thus, eleven patients were included in the study.  Table 1 shows spirometry for included and excluded patients. We did not identify statistically significant differences in relation to spirometry values (Table 1).

Eleven patients were included in this protocol. All patients were receiving oral corticosteroids, inhaled bronchodilators, and antibiotics. According to FEV_1_ values, 18.1% were classified as moderate, 45.4% severe, and 36.3% very severe COPD. The BODE index showed that 27.2% were in class II, 27.2% in class III, and 45.4% in class IV. Nutritional depletion was identified in 27.3% of patients.  Table 2 shows patient characteristics.

Table 3 shows patient characteristics before and after aerobic exercise. All patients completed 15 minutes of aerobic exercise. We observed a significant increase in systolic blood pressure (125.2 ± 13.6 versus 135.8 ± 15.0; *p* = 0.004) and respiratory rate (20.9 ± 4.4 versus 25.2 ± 4.5; *p* = 0.008), but neither of those parameters was considered clinically significant. There was a statistically significant decrease in SpO_2_ (93.8 ± 2.3 versus 88.5 ± 5.7; *p* = 0.001) even with supplemental O_2_, offered to all patients during exercise, and increased dyspnea index after the activity. Mean speed was 1.9 km/h with 0.7° slope during aerobic exercise. None of the patients showed arrhythmia or thoracic or leg pain (Table 3).

## 5. Discussion

This study evaluated the clinical effects of aerobic exercise during hospitalization in exacerbated COPD patients. We did not identify any clinically significant severe adverse events during the aerobic exercise.

Most patients (54.5%) had severe dyspnea according to the Borg scale and none felt fatigue in the legs. Our results agree with those of Troosters et al. (2010) who performed anaerobic training with 17 hospitalized COPD patients and did not observe adverse clinical events. However, a randomized study of hospitalized COPD patients was performed by Tang et al. (2012) using three groups: control (*n* = 11), low-intensity exercise (*n* = 11), and moderate-high intensity exercise (*n* = 10). The low-intensity training group performed aerobic training each day for 7.5 minutes at 40% of the 6-minute walk test speed associated with muscle training at a load of 40% of one maximum repetition. The moderate-high intensity training group performed the same activities, but with a load of 70%. The authors identified 13 adverse events; one was a serious arrhythmia event in patients during mild training. The most frequently reported adverse event in the study was the feeling of fatigue or malaise which was more frequent in the moderate-high intensity group, but without significant difference [19].

Another point related to adverse clinical events was the beginning of exercise training after hospitalization. Our study showed no negative clinical effects beginning after 72 hours of hospitalization. We did not find any recommendations in literature about the right time to initiate aerobic training during hospitalization [9]. There are careful recommendations for initiating training in the 48 hours after admission. For instance, Greening et al. [20] evaluated the muscular rehabilitation associated with aerobic exercise and early neuromuscular stimulation in hospitalized COPD patients. They found no difference between intervention and control groups in hospital readmission rate. However, there was an increased mortality risk after 12 months in patients who received intervention during hospitalization. On the other hand, other studies which began exercise after 48 hours hospitalization showed no negative outcomes, but those studies did not follow patients for more than one month [21, 22].

Our patients presented more severe obstruction (FEV1 34%) than those in literature. In Troosters et al. (2010), FEV_1_ values were on average 50% in the control group and 40% in the intervention group, similar to Ali et al. [23], whose FEV_1_ values were 44% in the control group and 46% in the intervention group. The mean 6MWD for our patients was lower than in literature; Troosters et al. (2010) and Borges and Carvalho (2014) showed that patients walked almost 100 metres more than our patients. Our patients presented more severity than those in literature hospitalized with exacerbated COPD.

The aerobic exercise protocol performed in this study is clinically safe for hospitalized COPD patients with the same characteristics. The predominant exclusion criteria were the presence of a comorbidity that limited mobility and high dyspnea score. Therefore, the safety of aerobic exercise cannot be generalized for all COPD patients with exacerbation.

## 6. Conclusion

Aerobic exercise conducted after 72 hours of hospitalization in patients with exacerbated COPD appears to be safe, as we observed no adverse clinical events during or after exercise.

## Figures and Tables

**Table 1:** Comparison between included and excluded patients performing short duration physical activity.

*N* = 59	Excluded	Included	*p*
	*N* = 48	*N* = 11	
Age, y	71.6 ± 11.8	65.5 ± 11.1	0.05
FEV1, L	0.99 ± 0.49	0.68 ± 0.35	0.25
FEV1, %	44.3 ± 16.3	34.2 ± 13.9	0.25
FEV1/FVC	0.49 ± 0.10	0.43 ± 0.05	0.10

Values expressed as mean ± standard deviation. FEV1: forced expiratory volume in one second; FVC: forced vital capacity. *p* < 0.05, comparisons evaluated by *t*-test.

**Table 2:** Characteristics of the 11 patients hospitalized with exacerbated COPD.

	*N* = 11
Female, %	63.6
Age, y	65.5 ± 11.1
Smoking (pack-years)	56.3 ± 39.9
FFMI, Kg/m^2^	16.1 ± 2.16
BMI, Kg/m^2^	26.0 ± 4.72
BODE index	6.0 ± 1.7
Charlson index	3.2 ± 2.0
6MWD, m	224.8 ± 114.2

Values expressed as mean ± standard deviation. All patients were receiving oral corticosteroids, inhaled bronchodilators, and antibiotics. FFMI: fat free mass index; BMI: body mass index; BODE: body mass index, airflow obstruction, dyspnea, and exercise capacity; 6MWD: six-minute walk distance.

**Table 3:** Clinical characteristics of patients in the intervention group during physical activity on the first day of hospitalization.

*N* = 11	Start of test	End of test	*p*
	*N* = 11	*N* = 11	
SBP, mmHg	125.2 ± 13.6	135.8 ± 15.0	**0.004**
DBP, mmHg	80.3 ± 9.3	89.6 ± 15.1	0.205
RR, rpm	20.9 ± 4.4	24.2 ± 4.5	**0.008**
HR, bpm	99.2 ± 11.5	119.1 ± 11.1	0.092
SpO_2_, %	93.8 ± 2.3	88.5 ± 5.7	**<0.001**
BORG	1.2 ± 1.3	5.0 ± 2.9	**<0.001**
BORG LL	0.3 ± 1.1	2.3 ± 2.7	0.106

Values expressed as mean ± standard deviation. PA deviation: SBP: systolic blood pressure mmHg; DBP: diastolic blood pressure; HR: heart rate per minute; RR: respiratory rate per minute; SpO_2_: peripheral oxygen saturation; BORG: sensation of dyspnea; BORG LL: feeling of fatigue. Statistical analysis by paired *t*-test.
